# Cancer-control outcomes of Radium- 223-pretreated lutetium- 177-PSMA Radioligand vs. Radium- 223-naïve mCRPC patients

**DOI:** 10.1007/s00259-025-07256-5

**Published:** 2025-04-07

**Authors:** Mike Wenzel, Lena Theissen, Daniel Groener, Maximilian Kriegmair, Markus Graefen, Tobias Maurer, Georg Salomon, Benedikt Hoeh, Carolin Siech, Severine Banek, Felix K. H. Chun, Philipp Mandel

**Affiliations:** 1 Department of Urology, Goethe University Hospital, Frankfurt, Frankfurt/Main, Germany; 2 Department of Nuclear Medicine, Goethe University Hospital Frankfurt, Frankfurt, Germany; 3 Urologische Klinik München Planegg, Planegg, Germany; 4 Department of Urology and Urosurgery, University Medical Center Mannheim, Medical Faculty Mannheim, Heidelberg University, Mannheim, Germany; 5 Martini-Klinik Prostate Cancer Center, University Hospital Hamburg-Eppendorf, Hamburg, Germany; 6 Department of Urology, University Hospital Hamburg-Eppendorf, Hamburg, Germany

**Keywords:** MCRPC, PFS, OS, Survival, Lu- 177, Ra- 223

## Abstract

**Purpose:**

Radium- 223 and Lutetium- 177 prostate-specific membrane antigen radioligand therapy (Lu- 177-PSMA) are approved for the treatment of patients with metastatic castration-resistant prostate cancer (mCRPC). Data on cancer-control outcomes of sequential therapy of Lu- 177-PSMA after radium- 223 are rare.

**Methods:**

Using the Frankfurt Metastatic Cancer database of the Prostate (FRAMCAP) database, we analyzed progression-free (PFS) and overall (OS) survival of patients after radium- 223 pretreatment vs. radium- 223-naïve controls undergoing Lu- 177-PSMA radioligand within 1 st- 7 th line mCRPC treatment.

**Results:**

Of 329 Lu- 177-PSMA mCRPC patients 19% were radium- 223 pretreated, while 81% radium- 223-naïve. The median number of administered mCRPC systemic treatment administrations were significantly higher for radium- 223 pretreated patients (4 vs. 3, *p* < 0.01). No difference in further baseline or cancer characteristics were observed, similar to PSA response under Lu- 177-PSMA treatment. In PFS analyses, no significant difference between radium- 223 pretreated vs. radium- 223-naïve Lu- 177-PSMA mCRPC patients were observed, with median PFS of 16 vs. 12 months (hazard ratio [HR]: 0.73, confidence interval [CI]: 0.52–1.02, p = 0.063). In OS analysis, also no significant differences were observed with median OS of 18 vs. 15 months for radium- 223 pretreated vs. radium- 223-naïve Lu- 177-PSMA mCRPC patients (HR: 0.99, CI: 0.71–1.37, p > 0.9). Finally, after additional multivariable adjustment, no differences in PFS and OS outcomes between both groups were observed.

**Conclusion:**

Sequential treatment with radium- 223 prior to Lu- 177-PSMA does not affect PFS or OS outcomes in mCRPC patients. Therefore, this real-world cohort suggests that both radiopharmaceuticals can be administered within mCRPC treatment algorithm.

## Introduction

Through significant advances in the treatment of metastatic castration resistant prostate cancer (mCRPC) within the last decades, a range of different therapeutic options are nowadays available [1–3]. One therapeutic option concerning bone metastases is the use of radium- 223 dichloride (radium- 223). Radium- 223 specifically targets bone metastases, and the therapy involves short-range internal radiation, primarily from alpha emitters [3–5]. The initial European Medical Agency (EMA)-approval based on ALSYMPCA trial in 2013 showed improved overall survival (OS) in men with mCRPC and bone metastases [5–7]. Conversely, more recent data from the Era- 223 study in 2019, in which the combined treatment of abiraterone plus radium- 223 was administered in mCRPC patients, an increased rate of bone fractures were observed [8]. Since Era- 223, the use of radium- 223 was viewed critically by many practicing physicians and is now only used to a limited extent in clinical practice and is now restricted to advanced mCRPC lines for heavily pretreated patients. However, a second clinical trial showed some discrepancy to that, since data from the contemporary available PEACE- 3 trial in 2024 showed that the combination of radium- 223 plus enzalutamide prolongs radiographic progression-free survival (PFS) in mCRPC with bone metastases, resulting in a significant increased PFS and OS rate, compared to enzalutamide alone [9].

The VISION trial demonstrated that Lu- 177-PSMA prolongs PFS and OS, leading to EMA approval, and it is now frequently applied as an early treatment strategy in mCRPC [10–14]. Radium- 223 and Lutetium- 177 prostate-specific membrane antigen radioligand therapy (Lu- 177-PSMA) are both forms of internal radiation therapy; however, they differ fundamentally in their physical properties, mechanisms of action, and biological effects [10, 15]. While Lu- 177-PSMA selectively targets PSMA-expressing tumor cells and delivers beta radiation, Radium- 223 is a bone-seeking alpha emitter that predominantly affects osteoblastic metastases. These differences result in distinct microdosimetry, dose rates, and targeting strategies. Given that both therapies are forms of internal radiation, there may be concerns that patients undergoing both treatments could exhibit lower response rates. However, their distinct targeting suggests complementary rather than redundant effects. Currently only a few data on patients who were treated sequentially with radium- 223 and subsequently with Lu- 177-PSMA are available [16–20].

We addressed this void and relied on the Frankfurt Metastatic Cancer Database of the Prostate (FRAMCAP), to compare cancer control outcomes such as PFS and OS in radium- 223-pretreated vs. radium- 223-naïve Lu- 177-PSMA mCRPC patients. We hypothesized that clinical and statistical relevant cancer control differences may occur in men pretreated with radium- 223 and subsequent Lu- 177-PSMA therapy, relative to radium- 223-naïve Lu- 177-PSMA mCRPC patients.

## Materials and methods

### Study population

After obtaining approval from the local ethics committee (reference number: SUG- 5–2024) and in adherence to the principles of the Declaration of Helsinki, we performed a retrospective analysis of all mCRPC patients documented in the prospectively maintained FRAMCAP database. Notably, the FRAMCAP database includes all metastatic prostate cancer patients who underwent treatment at the Department of Urology at the University Hospital Frankfurt and discussed within a multidisciplinary tumor board since 2014. Inclusion criteria for the current study consisted of mCRPC patients aged over 18 who had to have undergone either at least one cycle of Lu- 177-PSMA radioligand treatment in the time period between 2014–2025 [20]. Stratification was made according to radium- 223 pretreatment, resulting in 329 eligible mCRPC patients for further analyses, of which 63 received initially radium- 223 and subsequent Lu- 177-PSMA.

### Radium- 223 and Lu- 177-PSMA radioligand therapy

Radium- 223 is administered intravenously every fourth weeks, though the schedule may be adjusted at the physicians’ discretion or treatment suspension due to hematological toxicity [20]. Indication for the administration of radium- 223 was symptomatic, exclusively bone metastases (lymph nodes not larger then 3 cm), based on conventional or PSMA-PET/CT staging. Radium- 223 was administered from first-line mCRPC treatment (period prior Era- 223 study) or from third-line mCRPC (after Era- 223 study results).

Therapy with Lu- 177-PSMA was also given intravenously every 6–8 weeks carried out by at the department of nuclear medicine. The prerequisite for therapy was a PSMA-positive lesion in PSMA-PET/CT, as described previously [20–23]. Lu- 177-PSMA was administered from first-line mCRPC (as individual compassionate use after previous multidisciplinary team discussion) until 7 th line after previous androgen receptor pathway inhibitor (ARPI) and taxane-based chemotherapy for mCRPC. The current analyses focused solely on cancer-control outcomes; toxicity rates were recently published by our working group [20].

### Statistical analysis

Descriptive statistics included the computation of frequencies and proportions for categorical variables used in the analysis. Median values and interquartile ranges (IQR) were reported for continuous variables. Statistical significance for differences in proportions was assessed using the Chi-square test, while the t-test and Kruskal–Wallis test were employed to evaluate differences in distributions.

The primary endpoints of the study were PFS and OS outcomes of radium- 223 pretreated vs. radium- 223-naïve Lu- 177-PSMA mCRPC patients in Kaplan–Meier estimates, relying on log-rank tests. PFS was defined as the time between the start of therapy with Lu- 177-PSMA until the start of another systemic line of therapy due to clinical or radiographic progression, treatment withdrawal or death. OS was defined as the start of Lu- 177-PSMA treatment until death.

Uni- and multivariable adjusted Cox regression models were used for PFS and OS analyses, to test the influence of radium- 223-pretreatment in Lu- 177-PSMA mCRPC patients. Covariables included age at metastatic disease, Gleason score, Eastern Cooperative Oncology Group (ECOG) performance status, and De novo metastatic hormone-sensitive prostate cancer (mHSPC), as well as CHAARTED high volume mHSPC. For PFS analyses, further adjustment for the specific treatment line of Lu- 177-PSMA was made (first to seventh line mCRPC). For OS analyses, further adjustment for total number of received mCRPC lines was performed. All tests were two sided with a level of significance set at *p* < 0.05. R software environment for statistical computing and graphics (version 3.4.3) was used for all analyses.

## Results

### Study populations’ characteristics

A total of 329 Lu- 177-PSMA mCRPC patients were included in the current study of which 19% (n = 64) were radium- 223 pretreated vs. 81% (n = 265) were radium- 223-naïve (Table 1). Median time interval between radium- 223 and Lu- 177-PSMA treatment was 15 months. The median patient age at mCRPC diagnosis was 71 years (IQR:64–76), with a median PSA level at the castration-resistant stage of 16 ng/ml (IQR:5–59 ng/ml). Patients has ECOG performance status of 0 in 53% vs. ECOG 1 in 40% vs. ECOG 2 in 6,8%. Local treatment to the prostate was carried out in 42% of all patients. Initial diagnosis of a de novo metastatic disease was 43%. When diagnosed with mHSPC, 64% of patients had high-volume disease. The median number of completed systemic treatments for mCRPC was three (IQR:2–5). Most mCRPC patients (80%) harbored bone metastases (± lymph nodes) vs. 10% lymph nodes only vs. 9.4% visceral metastases. Median PSA response during Lu- 177-PSMA treatment was 20% (IQR:0–66%). Median number of administered cycles were five (IQR: 3–6) for radium- 223 and four (IQR: 2–6) for Lu- 177-PSMA.

**Table 1:** Characteristics of 329 metastatic castration resistant prostate cancer (mCRPC) patients receiving 177-lutetium prostate-specific membrane antigen (Lu- 177-PSMA) radioligand therapy stratified according radium- 223 naïve vs. radium- 223 pretreatment

Variable	N	Overall *N* = 329^*1*^	Radium- 223 naïve, *N* = 265 (81%)^*1*^	Radium- 223 pretreated *N* = 64 (19%)^*1*^	*p*-value^*2*^
Age metastatic disease, yrs	315	69 (62, 74)	70 (63, 75)	68 (60, 74)	0.10
Age at mCRPC, yrs	220	71 (64, 76)	71 (65, 76)	69 (61, 76)	0.2
PSA at mCRPC, ng/ml	148	16 (5, 59)	16 (5, 46)	36 (6, 79)	0.3
Systemic treatment lines for mCRPC	329	3 (2, 5)	3 (2, 4)	4 (3, 5)	0.004
ECOG status	191				0.7
0		102 (53%)	74 (52%)	28 (56%)	
1–2		89 (47%)	67 (48%)	22 (44%)	
Cardiovascular disease	198	70 (35%)	63 (36%)	7 (30%)	0.6
Gleason score 8–10	281	201 (72%)	169 (73%)	32 (65%)	0.3
Local therapy (RP/RT)	329	138 (42%)	110 (42%)	28 (44%)	0.7
High volume mHSPC	123	79 (64%)	66 (62%)	13 (76%)	0.3
De Novo mHSPC	320	184 (58%)	146 (57%)	38 (59%)	0.7
PSA response under Lu- 177-PSMA, %	59	20 (0, 66)	17 (0, 51)	56 (0, 68)	0.3
PSA 50	59	19 (32%)	12 (26%)	7 (54%)	0.092
PSA 90	59	7 (12%)	5 (11%)	2 (15%)	0.6
Cycles radium- 223	60		5 (3, 6)		
mCRPC treatment line radium- 223	63				
1 st line				24 (38%)	
2nd line				17 (27%)	
3rd line				15 (24%)	
4 th line				6 (9.5%)	
5 th line				1 (1.6%)	
Cycles Lu- 177-PSMA	294	4 (2,6)	4 (2, 6)	4 (2, 6)	0.08
mCRPC treatment line Lu- 177-PSMA	324				
1 st line		28 (8.6%)	28 (11%)	0 (0%)	
2nd line		73 (23%)	57 (22%)	16 (26%)	
3rd line		113 (35%)	97 (37%)	16 (26%)	
4 th line		40 (12%)	27 (10%)	13 (21%)	
5 th line		44 (14%)	36 (14%)	8 (13%)	
6 th line		19 (5.9%)	13 (4.9%)	6 (9.8%)	
7 th line		7 (2.2%)	5 (1.9%)	2 (3.3%)	
Metastatic sides at mCRPC	138				0.2
M1a		14 (10%)	13 (11%)	1 (4.8%)	
M1b		111 (80%)	91 (78%)	20 (95%)	
M1c		13 (9.4%)	13 (11%)	0 (0%)	
First line mCRPC treatment	329				
ADT mono		28 (8.5%)	20 (7.5%)	8 (13%)	
Chemotherapy		61 (19%)	50 (19%)	11 (17%)	
ARPI		185 (56%)	164 (62%)	21 (33%)	
Lu- 177-PSMA		28 (8.5%)	28 (11%)	0 (0%)	
PARPi		2 (0.6%)	2 (0.8%)	0 (0%)	
Radium		24 (7.3%)	0 (0%)	24 (38%)	
None/Other/NA		1 (0.3%)	1 (0.4%)	0 (0%)	
^*1*^ Median (Q1, Q3); n (%)					
^*2*^ Wilcoxon rank sum test; Fisher’s exact test; Pearson’s Chi-square test					

Abbreviations: *PSA* Prostate-specific antigen, *ECOG* Eastern Cooperative Oncology group, *mHSPC* metastatic hormone-sensitive prostate cancer, *ADT* Androgen deprivation therapy, *ARPI* Androgen receptor pathway inhibitor, *PARPi* Poly adenosine diphosphate ribose polymerase inhibitor

### Characteristics of Radium-pretreated vs. Radium-naïve Lu- 177-PSMA patients

In comparison between radium- 223 pretreated vs. radium- 223-naïve Lu- 177-PSMA mCRPC patients (Table 1), patients with radium- 223 pretreatment harbored significantly more systemic treatment lines for mCRPC, relative to radium- 223-naïve patients (4 vs. 3, *p* < 0.01). No further statistically significant differences were observed in further baseline patient or cancer characteristics. Despite not reaching significance, radium- 223-pretreated mCRPC patients achieved higher PSA response (56% vs. 17% (p = 0.3). Lu- 177-PSMA was most frequently administered within third line mCRPC treatment in both radium- 223 pretreated vs. radium- 223-naïve groups (26% vs. 37%). In radium- 223 pretreated patients, radium- 223 was most frequently administered within the first-line mCRPC (38%).

### Progression-free survival: Radium-pretreated vs. Radium-naïve Lu- 177-PSMA mCRPC patients

**Fig. 1:**
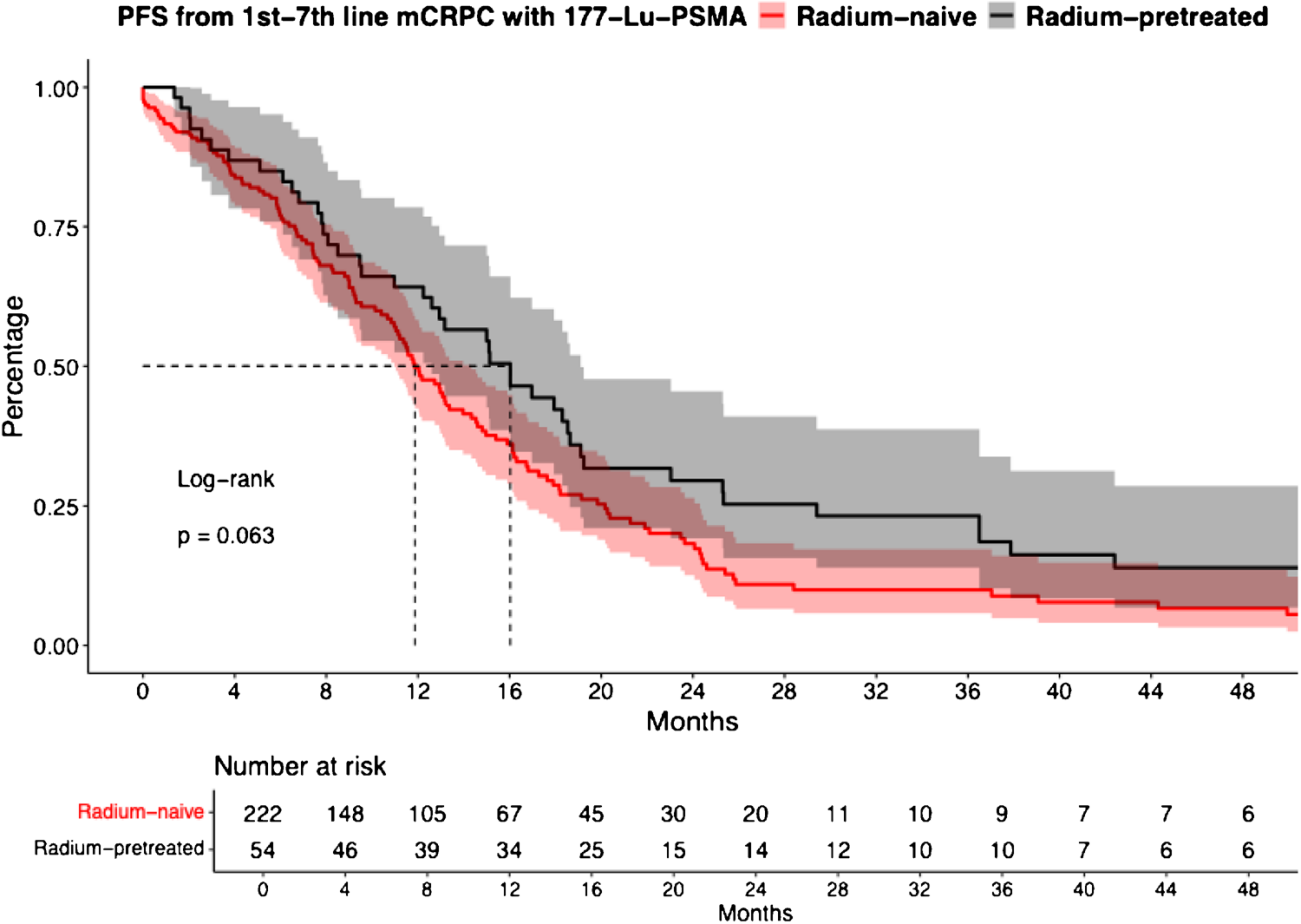
Kaplan Meier curves depicting progression-free survival (PFS) rates and confidence intervals in first to seventh-line metastatic castration-resistant prostate cancer (mCRPC) patients receiving lutetium- 177 prostate-specific membrane antigen (Lu- 177-PSMA) radioligand therapy and stratified according to radium- 223-naïve vs. radium- 223-pretreatment

In PFS analysis between mCRPC radium-naïve vs. radium-pretreated Lu- 177-PSMA patients, no significant difference was observed (Fig. 1, hazard ratio (HR): 0.73, confidence interval [CI]: 0.52–1.02, p = 0.063), with median PFS of 11.9 vs. 16.0 months for radium-naïve vs. radium-pretreated Lu- 177-PSMA mCRPC patients. Median 12- and 24-months PFS rates were 49.7% vs. 64.2% and 18.2% vs. 29.5% for radium-naïve vs. radium-pretreated Lu- 177-PSMA patients, respectively. After further multivariable adjustment for baseline patient and tumor characteristics, also no differences between both groups were observed (Table 2A, HR: 0.54, CI: 0.25–1.16, p = 0.11).

**Table 2:** Univariable und multivariable Cox regression models predicting progression-free survival (PFS; A) and overall survival (OS; B) in metastatic castration resistant prostate cancer (mCRPC) patients receiving Lutetium- 177 prostate-specific membrane antigen (Lu- 177-PSMA) radioligand therapy

	Univariable			Multivariable		
**(A) PFS**	**HR**	**CI**	**p value**	**HR**	**CI**	***p* value**
**Radium-naive**	**Ref**	**-**	**-**	**Ref**	**-**	**-**
Radium-pretreatment	0.73	0.52–1.02	0.063	0.54	0.25–1.16	0.11
**(B) OS**	**HR**	**CI**	**p value**	**HR**	**CI**	**p value**
**Radium-naive**	**Ref**	**-**	**-**	**Ref**	**-**	**-**
Radium-pretreatment	0.99	0.71–1.37	> 0.9	0.45	0.18–1.11	0.085

Abbreviation: *HR* Hazard Ratio, *CI* Confidence interval, *ECOG* Eastern Cooperative Oncology Group

Adjustment in multivariable Cox regression models for:

Age at metastatic disease, Gleason Score (6–7 vs. 8–10), synchronous vs. metachronous mHSPC, high volume mHSPC (vs. low volume), ECOG status (0 vs. 1–2)

For PFS further adjustment for number of treatment line (1 st to 7 th) and for OS number of received CRPC treatment lines was made.

### Overall survival: Radium-naïve vs. Radium-pretreated Lu- 177-PSMA mCRPC patients

**Fig. 2:**
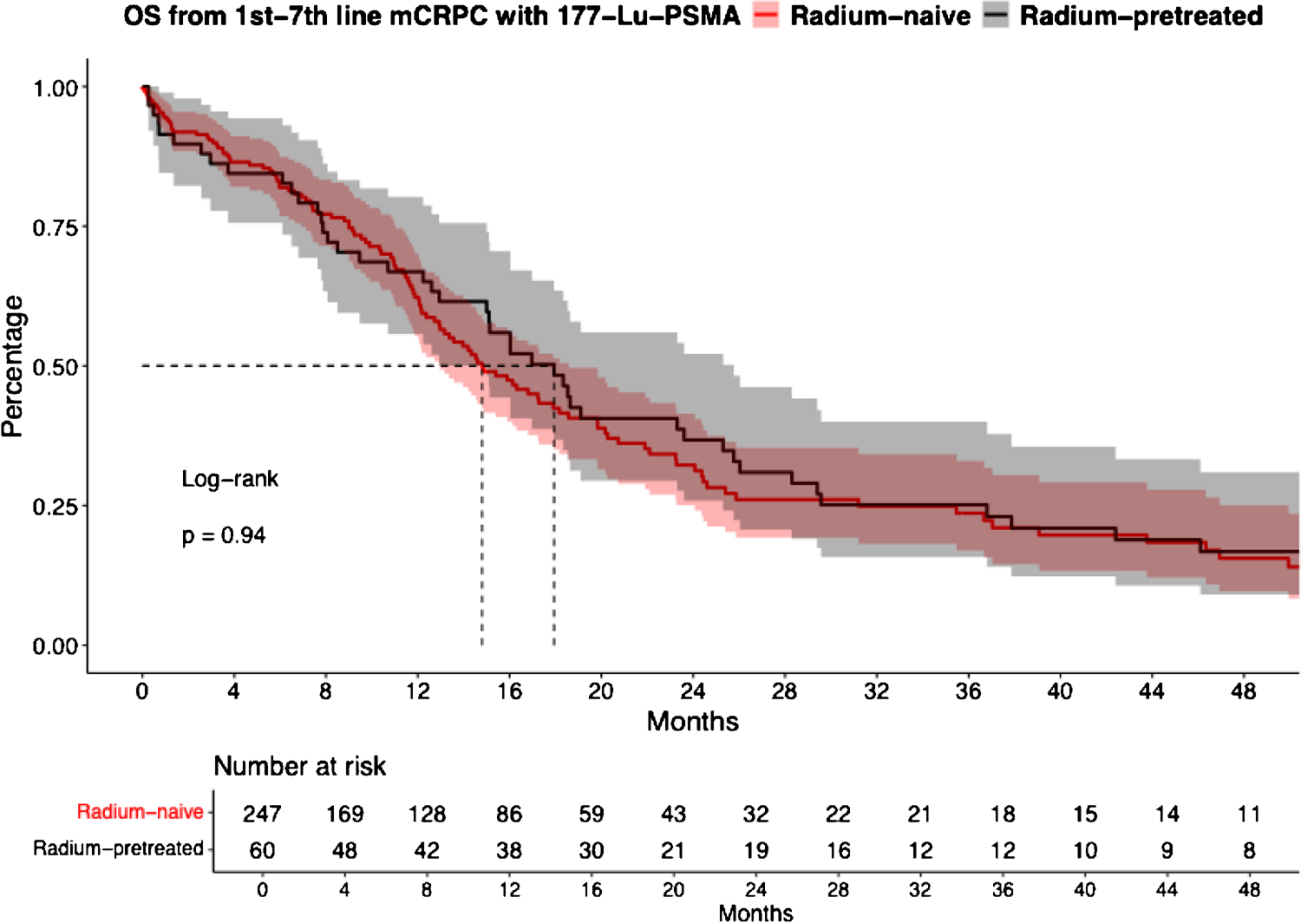
Kaplan Meier curves depicting overall survival (OS) rates and confidence intervals in first to seventh-line metastatic castration-resistant prostate cancer (mCRPC) patients receiving 177-lutetium prostate-specific membrane antigen (177-Lu-PSMA) radioligand therapy and stratified according to radium- 223-naïve vs. radium- 223-pretreatment

In OS analysis between mCRPC radium-naïve vs. radium-pretreated Lu- 177-PSMA patients, also no significant differences were observed (Fig. 2, HR: 0.99, CI: 0.71–1.37, *p* > 0.9), with median OS of 14.8 vs. 17.9 months for radium-naïve vs. radium-pretreated Lu- 177-PSMA patients. Median 12- and 24-months OS rates were 62.3% vs. 66.8% and 32.2% vs. 36.7% for radium-naïve vs. radium-pretreated Lu- 177-PSMA patients, respectively. After additional further multivariable adjustment in Cox regression models, also no differences between both groups were observed (Table 2B, HR: 0.45, CI: 0.18–1.11, p = 0.084).

## Discussion

We initially hypothesized that significant differences in baseline characteristics and cancer control outcomes might exist between mCRPC patients pretreated with radium- 223 and those who were radium-naïve before receiving Lu- 177-PSMA therapy. To test these hypotheses, we conducted a real-world study based on the FRAMCAP database, and our analysis revealed several important findings.

First, concerning baseline patient and tumor characteristics we found certain similarities between radium-naïve vs. radium pretreated groups receiving Lu- 177-PSMA. Although radium- 223 pretreated patients harbored significantly more systematic treatment lines for mCRPC and radium- 223 was most frequently administered within the first-line mCRPC (38%) no further statistically significant differences were observed, despite a meaningful higher PSA response for radium- 223-pretreated patients. One explanation may be that radium- 223 was available as an additional therapeutic option for patients with bone metastases only and that many of these patients were treated with radium- 223 before increasing concerns about radium- 223 therapy existed. Since mCRPC patients receive on average only 1–2 systemic treatment lines, further investigating additional sequential treatments that do not affect cancer-control outcomes is crucial to avoid potential undertreatment [2, 24].

Second, when cancer-control outcomes between radium- 223 naïve vs. radium- 223 pretreated Lu- 177-PSMA mCRPC patients were compared, also important observations were made. Specifically in PFS comparison, PFS did not differ in patients pretreated with radium- 223 (11.9 vs. 16.0 months for radium-naïve vs. radium-pretreated Lu- 177-PSMA, HR: 0.73, CI: 0.52–1.02, p = 0.063). These findings may reflect differences in tumor characteristics such as the higher rate of visceral metastases (11% vs. 0%) and the significantly lower number of received systemic treatments in the radium-naïve cohort [25]. Therefore, it is of great importance in the interpretation of the current study to rely on the further adjusted results for potential confounders in Cox regression models, which led to no statistical significance. These findings are particularly interesting, as the use of two internal radiotherapies in the interval have been often avoided within recent times, despite new evidence and suggestions such as from the US Prostate Cancer Conference [26–28]. Since the ERA- 223 study, radium- 223 has only been used to a limited extent in clinical practice. The results of the ERA- 223 study showed that the combined treatment of abiraterone acetate plus prednisone in mCRPC and radium- 223 led to an increased rate of bone fractures. Otherwise, the data from the PEACE- 3 trial showed that the combination of radium- 223 plus enzalutamide prolonged radiological PFS in mCRPC with bone metastases. The number of radiological disease progressions or deaths was significant lower in patients receiving the combination therapy compared to enzalutamide alone, probably leading to a more frequently use of radium- 223 in earlier mCRPC lines [8, 9]. Compared to previous publications, relative similarities can be found. For example, a multicenter report by Giannini et al., comparing 27 radium- 223-pretreated vs. 206 radium- 223-naïve Lu- 177-PSMA mCRPC patients found also comparable response rates between both groups [16]. Moreover, a more historical report from our department of nuclear medicine, relying on 28 radium- 223-pretreated mCRPC patients, receiving Lu- 177-PSMA within eight weeks after radium- 223, reported a median PFS of 10 months [20]. Similarly, a report by Ahmadzadehfar et al. also reported comparable treatment responses in 20 radium- 223-pretreated Lu- 177-PSMA mCRPC patients [29]. Finally in accordance with our findings, an additional study by Ahmadzadehfar et al. also reported no additional risk of lower OS with radium- 223 pretreatment [18].

Finally, when OS outcomes were compared, no significant difference in OS were observed between patients (14.8 vs. 17.9 months for radium-naïve vs. radium-pretreated Lu- 177-PSMA, HR: 0.99, CI: 0.71–1.37, p > 0.9). Similar findings were made after further multivariable adjustment in Cox regression models. These findings are important, since they provide robust findings of an administration of radium- 223 not being associated with reducing cancer-control outcomes of later Lu- 177-PSMA administration. Compared to previous studies, similar findings were made. The combination of radium- 223 followed by Lu- 177-PSMA was investigated in a multicenter study including by Rahbar et. al. relying on 133 patients, showed median OS of 13.2 months [17]. Conversely, the study by Giannini et al. found shorter OS in radium- 223-pretreated Lu- 177-PSMA patients [16].

Our study has limitations which need to be considered in its interpretation. First, patients underwent different numbers of treatment lines prior to radium and/or Lu- 177-PSMA administration, which may affect cancer-control outcomes and impact comparability with other studies. Moreover, some missing or unknown variables such as the exact number of bone metastases may have impacted primary endpoints. Second, the doses of Lu- 177-PSMA and radium- 223 administered may differ depending on the treatment protocol. Third, radium- 223 treatment was only used in patients with bone metastases, therefore some potential radium- 223 patients did not qualify for treatment with additional metastatic burden and may introduced some sort of selection bias. In addition, PSMA expression on PSMA-PET/CT was required for treatment with Lu- 177-PSMA, which may have also led to a potential selection bias. Finally, no data were available for potential side effects or adverse events or further laboratory values in this cohort. However, a recently report by our colleagues of the nuclear medicine department found an acceptable overall and mostly reversible incidence of hematologic side effects after Lu- 177-PSMA, even after radium- 223-pretreatment [20].

Taken together, we initially hypothesized that clinically meaningful and statistically significant cancer control differences may occur in mCRPC men pretreated with radium- 223 and subsequent Lu- 177-PSMA therapy, relative to radium- 223-naïve Lu- 177-PSMA mCRPC patients. Our study suggests that this sequential treatment, in particular the administration of radium- 223 prior to therapy with Lu- 177-PSMA, provides similar PFS and OS outcomes for Lu- 177-PSMA, relative to radium- 223-naïve patients. Therefore, our data supports the feasibility of using Lu- 177-PSMA even after radium- 223 pretreatment.

## Data Availability

The datasets generated during and/or analyzed during the current study are available from the corresponding author on reasonable request.
